# Complete genome sequence of the biocontrol yeast *Papiliotrema terrestris* strain LS28

**DOI:** 10.1093/g3journal/jkab332

**Published:** 2021-09-17

**Authors:** Davide Palmieri, Giuseppe Barone, Riccardo Aiese Cigliano, Filippo De Curtis, Giuseppe Lima, Raffaello Castoria, Giuseppe Ianiri

**Affiliations:** 1 Department of Agricultural, Environmental and Food Sciences, University of Molise, 86100 Campobasso, Italy; 2 Sequentia Biotech SL, Barcelona 08018, Spain

**Keywords:** *Papiliotrema terrestris*, biocontrol, genome sequencing, Tremellomycetes

## Abstract

*Papiliotrema terrestris* strain LS28 is a biocontrol agent selected for its antagonistic activity against several plant pathogens both in the field and postharvest. The availability of a genome sequencing sets the foundation for the identification of the genetic mechanisms of its antagonistic activity. The genome size is 21.29 Mbp with a G+C content of 58.65%, and genome annotation predicts 8,626 protein-encoding genes. Phylogenetic analysis based on whole-genome data confirms that *P. terrestris* is a Tremellomycetes more closely related to *Papiliotrema flavescens* than *Papiliotrema laurentii*.

## Introduction

The yeast strain LS28, previously described as *Papiliotrema laurentii* (ex *Cryptococcus laurentii*) and recently reclassified as *Papiliotrema terrestris* (Miccoli *et al.* 2020), is a biocontrol agent isolated from the apple fruit epiphytic microbiota and selected for its ability to counteract fungal pathogens of plants and fruits, both in-field and in the postharvest stage (Lima *et al.* 1998). As for many biocontrol agent yeasts, the main mechanism that underlines the antagonistic activity of *P. terrestris* is competition for nutrients and space. This mechanism relies on the ability of the biocontrol agent to rapidly adapt to and colonize fruit tissues through resistance to stresses generated in wounded fruit tissues, mainly oxidative stress, as demonstrated through chemical and genetic approaches (Castoria *et al.* 2003; Macarisin *et al.* 2010; Castoria *et al.* 2021). Subsequently, other mechanisms such as the production of extracellular β-1-3-glucanase that degrade fungal cell wall and the induction of host defense might have a role in its antagonistic activity (Castoria *et al.* 1997; Janisiewicz and Korsten 2002). The development of biocontrol and integrated methods to control fungal disease aims to reduce the necessity of chemical fungicides, because their extensive use has raised several ethical and technical concerns, such as the emergence of plant pathogens’ resistance as well as the health risks associated with the persistence of chemical residues in fruit, vegetables, and in the environment. It is now common practice to generate genome sequence and assembly as the foundation for the development of a microorganism as biocontrol agent because it allows its proper classification, patent protection, and the knowledge of its genetic potential that might be of crucial importance to exploit/upgrade its biocontrol activity.

In the present study, we generated a draft genome assembly of the biocontrol agent yeast *P. terrestris* strain LS28, a natural isolate that displayed elevated antagonistic activity against a wide range of plant pathogens both in the field and in postharvest.

## Materials and methods

### Sequencing and annotation of the *Papiliotrema terrestris* LS28 genome

*Papiliotrema terrestris* strain LS28 was grown in liquid YPD (Yeast extract 10 g L^−1^; Peptone 20 g L^−1^; Dextrose g L^−1^) at 28°C for 48 h, and genomic DNA was isolated using a CTAB extraction protocol (Pitkin *et al.* 1996). DNA sequencing was performed by Macrogen (Seoul, South Korea) using 150-bp paired end Illumina sequencing with Illumina Hiseq 2500. DNA-seq libraries were prepared using the TruSeq DNA PCR-free library kits following manufacturer’s instructions. Reads obtained were subjected to quality control before and after the trimming step using the software BBDuk v35.85 (http://jgi.doe.gov/data-and-tools/bb-tools/) (minimum quality 23 bp and minimum length 35 bp). Trimmed reads were subjected to K-mer content using KAT v2.4.2 (https://github.com/TGAC/KAT) with a K-mer size of 27 nucleotides to estimate ploidy and genome size. *De novo* assembly was performed with the software Spades v3.13.0 (Bankevich *et al.* 2012) with the following options –careful -k 39,49,59,69,79,99 –cov-cutoff auto. Scaffolding was carried out using the software SGA v1.0 (https://github.com/jts/sga/wiki/SGA-Design). Quality of the *de novo* assembly was assessed using QUAST v5.0.2 (https://sourceforge.net/projects/quast/), and by re-mapping trimmed reads on the assembly using BWA v0.7.17-r1188 (Li and Durbin 2010) and by assessing the quality of the mapping using Qualimap (http://qualimap.bioinfo.cipf.es/).

For RNA extraction, *P. terrestris* strain LS28 was grown in liquid YPD at 28° C for 48 h, and total RNA was isolated using a TRIzol protocol (Rio *et al.* 2010). RNA sequencing was performed at Genomix4life (Salerno, Italy) using 150 bp paired-end sequencing with Illumina Hiseq 2500. RNA-seq libraries were prepared using the TruSeq mRNA PCR-free library kits, following manufacturer’s instructions. Reads obtained were subjected to quality control and trimmed using the software BBDuk v35.85 (http://jgi.doe.gov/data-and-tools/bb-tools/) (minimum quality 15 bp and minimum length 35 bp). RNAseq reads were mapped against the *P. terrestris* strain LS28 assembly using STAR v2.6.1a in double pass mode (Dobin *et al.* 2013).

Genome annotation was performed with the Braker2 (v2.1.5) pipeline using a mapped .bam file as input and the option –fungus (Hoff *et al.* 2019). The quality of the annotation was assessed with BUSCO v4.0.2 (Simão *et al.* 2015), and a functional annotation was performed with the Pannzer2 pipeline (Toronen *et al.* 2018).

### Phylogenetic analysis of *Papiliotrema terrestris* LS28

For phylogenetic analysis, the predicted proteomes of representative basidiomycetes fungal species were downloaded from GenBank (last accessed on March 2021). In particular, within the Agarycomycotina, the representative genomes of the Agarycomycetes class, *Schizophyllum commune* strain H4-8 (accession GCF_000143185), *Laccaria bicolor* strain S238N-H82 (accession GCF_000143565), and *Coprinopsis cinerea* strain okayama7#130 (accession GCF_000182895) were selected. For the Tremellomycetes, which is the class of *Papiliotrema* species based on a previous classification (Liu *et al.* 2015), the following genomes were downloaded for phylogenetic analysis: *Tremella mesenterica* strain DSM 1558 (accession GCF_000271645), *Cryptococcus neoformans* var. *grubii* strain KN99 (accession GCA_002216725), *Kwoniella mangroviensis* strain CBS 8507 (accession GCF_000507465), *Phaffia rhodozyma* strain IPF (accession GCA_001007165), and *Cutaneotrichosporon oleaginosum* strain IBC0246 (accession GCF_001027345). Because only the genome assemblies were available for *P. laurentii* strain IF7SW-F4 (accession GCA_012922615), *P. laurentii* strain IF7SW-B5 (accession GCA_012922625), *P. laurentii* strain RY1 (accession GCA_000738825), and *Papiliotrema flavescens* strain NRRL Y-50378 (assembly GCA_000442785), the predicted proteomes of these strains were generated using GeneMark version 4.48_3.60_lic (Ter-Hovhannisyan *et al*. 2008) with the following options: –ES –max_intron 3000 –min_gene_in_predict 120 –fungus. For the Ustilaginomycotina, *Sporisorium reilianum* f. sp. *reilianum* strain SRS1_H2-8 (accession GCA_900162835), *Ustilago maydis* strain 521 (accession GCF_000328475), and *Malassezia sympodialis* strain ATCC 42132 (accession GCA_900149145) were selected, while for the Pucciniomycotina *Microbotryum lychnidis-dioicae* strain p1A1 Lamole (GCA_000166175), *Puccinia graminis* f. sp. *tritici* strain CRL 75-36-700-3 (accession GCF_000149925), and *Rhodosporidium kratochvilovae* strain LS11 (accession GCA_002917965.1) were selected. Last, *Saccharomyces cerevisiae* strain S288C (accession GCF_000146045) and *Neurospora crassa* strain OR74A (accession GCF_000182925) were used as outgroups.

In order to reconstruct the phylogenetic relationship of the species, the FASTA files of the predicted proteomes from the aforementioned species were analyzed with Orthofinder version 2.5.2 (Emms and Kelly 2019) with the following options: -M dendroblast -S diamond_ultra_sens. The STAG (Species Tree Inference form All Genes) method was used on 689 single copy conserved orthologous proteins.

## Results and discussion

**Figure 1:**
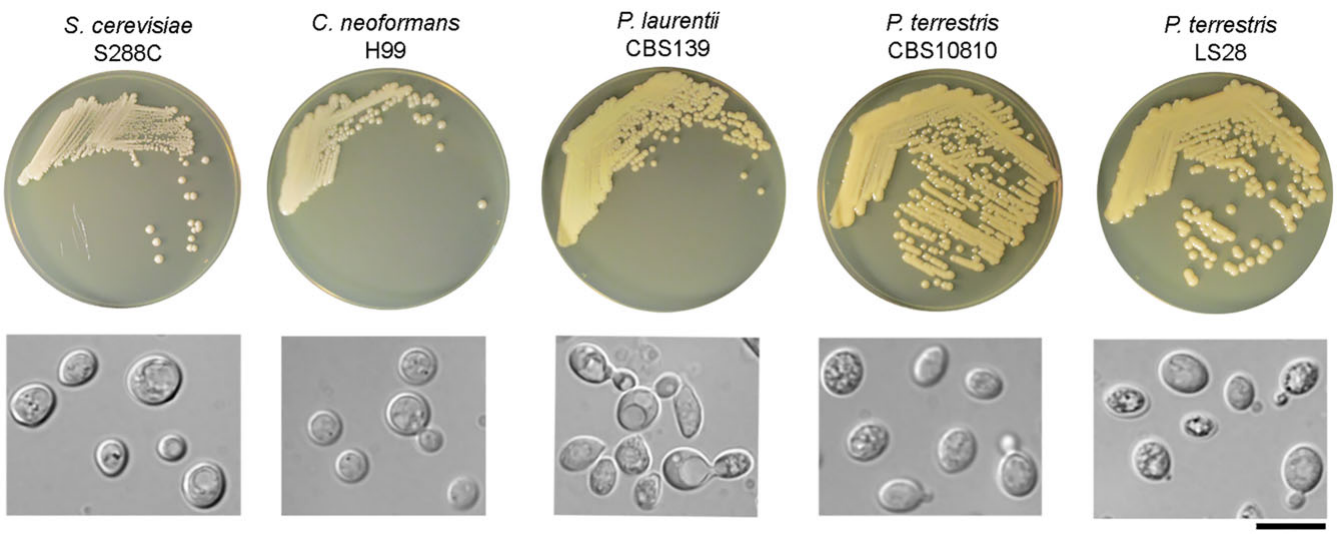
*Saccharomyces cerevisiae* strain S288C, *Cryptococcus neoformans* strain H99, Papiliotrema laurentii type strain CBS139, *Papiliotrema terrestris* type strain CBS10810, and *Papiliotrema terrestris* strain LS28 streaked onto YPD agar and incubated at 28°C for 3 days; photomicrography of cells is shown below each plate from which the cells were withdrawn for microscopy observations. Bar = 10 µm.

The genus *Papiliotrema* (class Tremellomycetes, order Tremellales, family Rhynchogastremaceae) includes a monophyletic clade of more than 20 yeasts (Liu *et al.* 2015). The majority of the *Papiliotrema* species were previously known as *Cryptococcus* but were reclassified for their closer phylogenetic relation with *P. bandonii* CBS 9107, the type species of the genus (Sampaio *et al.* 2002). *Papiliotrema* species are characterized by a saprophytic lifestyle and are typically environmental yeasts, being isolated from soil, but also from different plants, fruits, and flowers. The majority of the studies on *Papiliotrema* species regards *P. laurentii* (previously known as *C. laurentii*), which is often reported as a beneficial promising yeast in biological control of plant pathogens (Lima *et al.* 1998; Castoria *et al.* 2003; Qin and Tian 2005; Zhang *et al.* 2017; De Curtis *et al.* 2019). However, it was also reported as a human pathogen in immunocompromised patients [review in Londero *et al.* (2019)]. The latter finding might be due to the characteristics of the yeast itself as a basidiomycetes, which are characterized by high phenotypic plasticity as observed in closely-related *Cryptococcus* pathogenic species (*C. neoformans, C. deneoformans*, and *C. gattii*) (D'Souza *et al.* 2011; Li *et al.* 2012), or more simply it is the consequence of erroneous identification based only on biochemical tests, which cannot discriminate between very closely related species. This was the case of the *Papiliotrema* species under study in the present work, *P. terrestris* strain LS28; this yeast was previously classified as *P. laurentii* through API (Analytical Profile Index) test but was then reclassified as *P. terrestris* following a phylogenetic analysis of the ribosomal ITS and D1-D2 regions, and of the *TEF1* and* RPB1* genes (Miccoli *et al.* 2020). *Papiliotrema terrestris* looks very similar to *P. laurentii* on agar plate, both producing colonies that are yellowish, smooth and mucous, and at the microscope, with globose to ovoid cells, usually with monopolar asexual budding; large cellular organelles, likely vacuoles, could only be observed in *P. laurentii* under the growth conditions that were used (4 days in YPD agar at 30°C) (Figure 1). Both *Papiliotrema* species are clearly distinct from the model yeasts for ascomycetes and basidiomycetes *S. cerevisiae and C. neoformans*, respectively (Figure 1).

*Papiliotrema terrestris* is a saprophytic yeast that has been isolated for the first time from forest soil in Oklahoma, USA (Crestani *et al.* 2009). The strain of interest in this study is LS28, which was isolated from apples cv Limoncella (Larino, Italy). It is an important biocontrol agent that displayed high antagonistic activity against economically-relevant postharvest plant pathogens, such as *Penicillium expansum*, *Botrytis cinerea*, *Rhizopus stolonifer*, *Aspergillus niger*, and *Monilia fructigena* on different fruits (Lima *et al.* 1998; De Curtis *et al.* 2019; Castoria *et al.* 2021). Sequencing the genome of *P. terrestris* LS28 is crucial to pave the way to the identification of the genetic traits of its antagonistic activity, and for comparative genomics studies that might contribute to define genomic evolution within the Tremellomycetes.

Whole-genome sequencing of *P. terrestris* LS28 was performed by Macrogen using Illumina technology, with a starting data set of 28 million of paired-end reads. Trimmed reads (∼20.8 million) ranging from 35 to 150 bp were subjected to k-mer analysis that revealed low heterogeneity and an estimated genome size of 19.57 Mbp. Before proceeding to a *de novo* assembly, the reads were mapped against the genomes of *Cryptococcus amylolentus*, *Cryptococcus depauperatus*, *Kwoniella heveanensis*, *P. laurentii*, and *Cryptococcus neoformans*. Unfortunately, the percentage of mapped reads ranged from 0.08% (against *C. depauparatus*) to 1.3% (against *P. laurentii*), thus excluding the possibility to perform a reference guided assembly.

Therefore, a *de novo* genome assembly of Illumina-generated reads was performed using the software Spades, which resulted in the generation of 1622 contigs, covering 21.29 Mbp (108.7% of the predicted genome size). The largest contig measured 115 kbp and the N_50_ was 25 kbp; G+C content was 58.65%. A complete genome assembly statistic is provided in Table 1. Trimmed reads were mapped back against the genome assembly: ∼99.97% of the reads could be mapped on the assembly uniquely, with a mean coverage of 93× and an average mapping quality of 59, thus excluding large duplication events.

**Table 1:** Quast-generated statistical report of the genome assembly of *Papiliotrema terrestris* LS28

Assembly	*Papiliotrema terrestris*	*Papiliotrema terrestris*_broken
# contigs (≥0 bp)	1,622	–
# contigs (≥1000 bp)	1,382	1,402
# contigs (≥5,000 bp)	996	1,006
# contigs (≥10,000 bp)	687	691
# contigs (≥25,000 bp)	259	259
# contigs (≥50,000 bp)	67	61
Total length (≥0 bp)	21,299,962	–
Largest contig (bp)	115,014	115,014
GC content (%)	58.65	58.65
N50 (bp)	25,684	25,326
Number of protein-coding genes	8,626	8,626

All statistics are based on contigs of size ≥500 bp, unless otherwise noted [*e.g.*, “# contigs (≥0 bp)” and “Total length (≥0 bp)” include all contigs].

**Figure 2:**
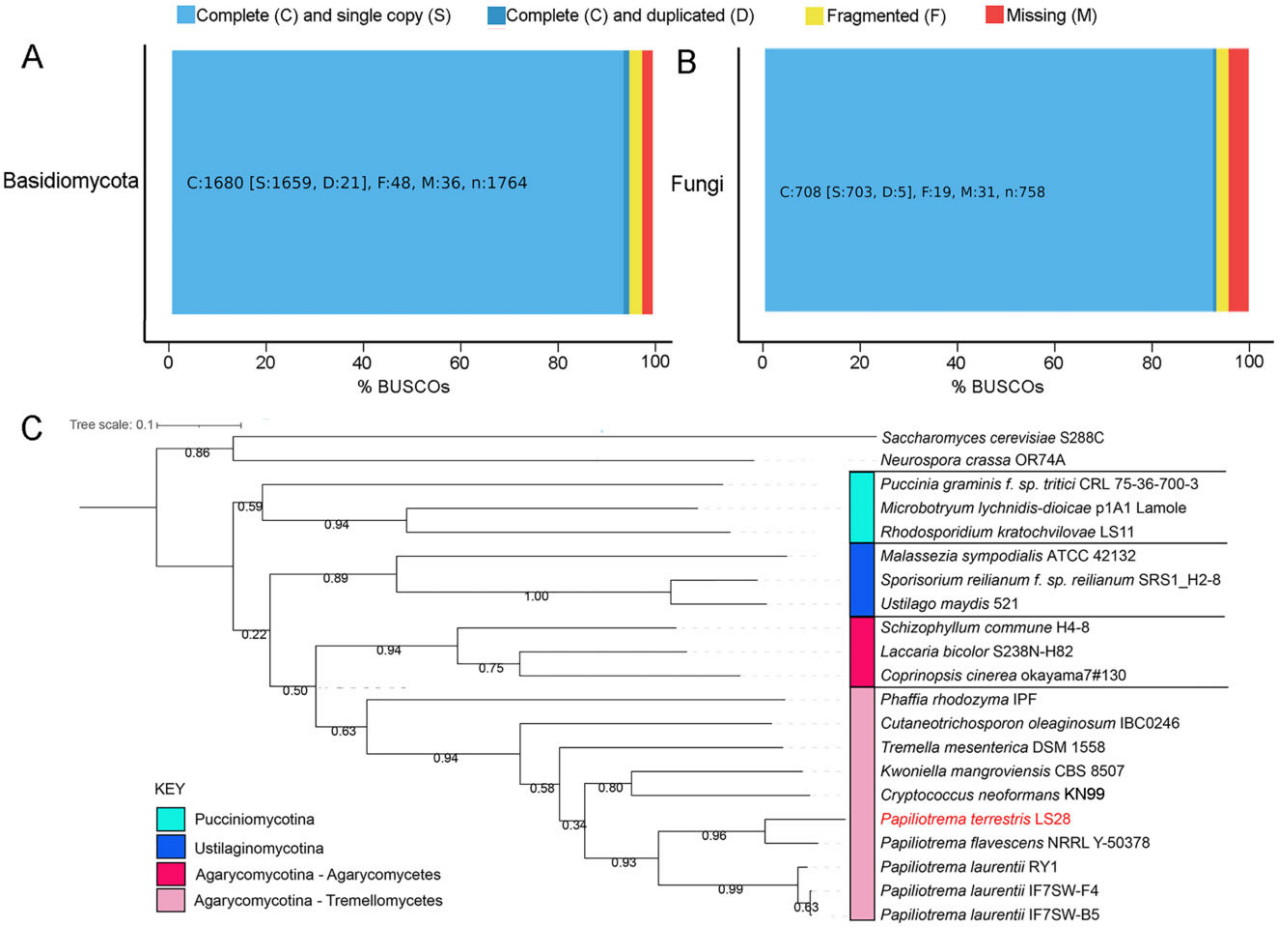
Graphical representation of the results of the BUSCO pipeline carried out on the predicted *Papiliotrema terrestris* proteins against Basidiomycota (A) and Fungi (B) databases. In C, the maximum likelihood tree of 689 single copy concatenated OrthoFinder groups is reported with the bootstrap values indicated.

For an accurate *ab initio* gene prediction and gene annotation, RNAseq evidences were used. For analysis of the RNAseq data, ∼88 millions of paired-end reads were generated and subjected to trimming, resulting in the production of 79,701,030 high-quality paired-end reads. More than 97% of these reads mapped to the genome assembly, indicating its high quality; about 96% of the reads mapped uniquely to the genome. Genome annotation generated 8,626 gene models. Analysis of the predicted proteins with BUSCO revealed high quality of the annotation, with more than 92% of the genes considered complete and as single copy using the fungi_odb10 dataset and more than 95% using the basidiomycota_odb10 dataset (Figure 2, A and B). Functional annotation revealed that a gene description could be associated to 6181 proteins, a Gene Ontology function to 7160 sequences, and a KEGG enzyme classification was associated to 4763 proteins. The genome size of ∼20 Mbp and the number of predicted genes reflect that of other Tremellomycetes yeasts, such as *Cryptococcus* spp and *Kwoniella* spp (Janbon *et al.* 2014; Sun *et al.* 2017; Passer *et al.* 2019). Phylogenetic analysis carried out on 689 single copy conserved orthologous proteins revealed the closely relation of *P. terrestris* and* P. flavescens*, and their phylogenetic distance from *P. laurentii* species (Figure 2C), thus confirming previous analyses carried out on ribosomal regions or single loci (Liu *et al.* 2015; Miccoli *et al.* 2020).

Understanding the genetic bases of antagonistic activity by biocontrol agents through insertional and random mutagenesis studies is the basic requirement to promote the utilization of biological (and integrated) control for the reduction of chemical fungicides. The genome presented in this study was used to generate *P. terrestris-*specific selective markers by cloning the hygromycin B and the neomycin sulphate G418-encoding genes under the control of the promoter and terminator of the histone *H3* gene. These cassettes were used to develop a targeted mutagenesis strategy that allowed to characterize the critical role of transcription factor Yap1 in biocontrol activity of *P. terrestris* LS28 against *P. expansum* and* M. fructigena* (Castoria *et al.* 2021). Besides functional genetics, the generated *P. terrestris* genome sequence will be used as a reference in transcriptomic analysis and will serve for expanding the availability of genomics data within the Tremellomycetes hence contributing to elucidate the evolutionary difference between pathogenic and non-pathogenic species.

## Data availability

Data reported in this study can be found on NCBI under Bioproject PRJNA744866. In particular, raw DNA and RNA reads are available in the Sequence Reads Archive (SRA) database (accessions SRR15684031 and SRR15684030, respectively). The Whole Genome Shotgun project has been deposited at DDBJ/ENA/GenBank under the accession JAHXHD000000000. The version described in this paper is version JAHXHD010000000.

## Funding

This work was supported by the PON AIM program Azione I.2 “Attrazione e Mobilità dei Ricercatori” (to G.I. and R.C.) (PON AIM AIM1804798).

## Conflicts of interest

The authors declare that there is no conflict of interest.
